# Towards Precision Medicine: Inclusion of Sex and Gender Aspects in COVID-19 Clinical Studies—Acting Now before It Is Too Late—A Joint Call for Action

**DOI:** 10.3390/ijerph17103715

**Published:** 2020-05-25

**Authors:** Evelyne Bischof, Sabine Oertelt-Prigione, Rosemary Morgan, Sabra L. Klein, The Sex and Gender in COVID19 Clinical Trials Working Group (SGC), Gender and COVID19 Working Group

**Affiliations:** 1 Department of Advanced Biomedical Sciences, Federico II University of Naples, Via Pansini, 5, 80131 Naples, Italy; bischofevelyne@gmail.com; 2 School of Clinical Medicine, Shanghai University of Medicine and Health Sciences, Shanghai 201318, China; 3 Women’s Brain Project, 8357 Guntershausen, Switzerland; 4 Department of Primary and Community Care, Radboud University Medical Center, 6525 Nijmegen, The Netherlands; sabine.oertelt@radboudumc.nl; 5 Institute of Legal and Forensic Medicine, Charité—Universitätsmedizin, 10117 Berlin, Germany; 6 Department of International Health, The Johns Hopkins Bloomberg School of Public Health, Baltimore, MD 21205, USA; Rosemary.Morgan@jhu.edu; 7 W. Harry Feinstone Department of Molecular Microbiology and Immunology, The Johns Hopkins Bloomberg School of Public Health, Baltimore, MD 21205, USA; 8 Department of Biochemistry and Molecular Biology, The Johns Hopkins Bloomberg School of Public Health, Baltimore, MD 21205, USA

The COVID-19 global pandemic is accelerating investigations for effective vaccines and repurposable validated therapeutics. Current data analyses strongly suggest that the disease mostly affects the elderly population and patients with pre-existing conditions [1,2]. Considerably less attention has been drawn towards the sex distribution of case fatalities, which is increasingly showing disparities in mortality rates that varies geospatially, and along socioeconomic factors [3,4]. Reported death estimates by sex vary greatly across contexts and population groups and may change over time. In addition, social factors, such as testing and reporting bias in females, or differences in exposure due to behavioral and risk differences, may play a role, e.g., due to comorbidities such as diabetes or differences in societal and gender norms. While the observed male dominance in COVID-19 prevalence and mortality across most countries and cultures may suggest a role for biological differences, the potential long-term impact of gender-related factors on mortality, especially in diverse socioeconomic contextS, cannot be underestimated [5,6].

The role of immunological differences between females and males in the responses to SARS-CoV-2 infection appears to be justified. There is ample evidence that antiviral immunity differs between the sexes [5]. These are caused by e.g., sex steroid hormone signaling (i.e., testosterone, estrogens, and progesterone), genetics (e.g., immune function genes that escape X inactivation), and sex-specific composition of the microbiome. Sex differences in immunosenescence and immune function not only impact immunity to viruses, but to vaccines and immunotherapies, as well [5,6,7]. In the context of SARS-CoV-2, these differences could impact susceptibility and initial response to the virus as well as choice of acute and long-term therapy of the COVID-19 pathology. In current and future trials for COVID-19, sex as a biological variable should be factored in and understood, along with the wider gendered implications of the COVID-19 crises, with the broader concept of how biological factors intersect with gendered differences in exposure, transmission, and socio-economic means. Consequently, the pandemic may not just lead to differences in disease susceptibility and manifestation between men, women, and people with non-binary identities, but also exacerbate unequal access to treatment and long-term vulnerabilities.

Given their non-negligible impact on health, sex and gender dimensions, along with other socio-economic stratifiers, need to inform the design, conduct, analysis, and reporting of current and forthcoming trials. Moving beyond sex-disaggregated data collection and including variables such as disability, age, ethnicity, migration status, socioeconomic status, or geographic location, will contribute to ensure health benefits from clinical trials for all. To better understand and respond to the burden posed by COVID-19, both on health systems and different segments of human populations, gender dimensions must be recognized as an intersecting component of wider structural inequalities [8]. Moreover, the COVID-19 pandemic is exposing, most acutely, the wider social inequalities that are based on gendered social, cultural, and economic faultiness, whether it is leaving a majority of frontline workers (in many contexts mostly women) without PPE, the disproportionate burden of unpaid care on women, or gender-based violence perpetuated within the household, apart from the economic devastations experienced by the poor and women in the poorest quintiles.

Equity in clinical trials starts with the consideration of both sex and gender dimensions in studies on novel and repurposed drugs [9,10]. Biomedical AI-researchers can assist in this effort to reconceptualize the human subgroups included for analysis, emphasizing the rigorous justification of exclusion and avoiding assumptions that may have serious implications in terms of generalizability of outcomes [9,11,12]. Ignoring aspects of sex and gender in data collection and analysis in clinical trials has had detrimental consequences in the past. Eight out of ten drugs withdrawn from the US market in the late 1990s had significantly more side effects in women than men, including fatal torsade-de-pointe after excessive QT interval prolongation. The, yet to be accurately tested, proposed therapeutic regimen of hydroxychloroquine and azithromycin for COVID-19 includes two QT-prolonging agents. Next to potential sex differences in side effects, gender-related aspects have to be considered. For example, despite the disproportionately high mortality of Ebola viral disease (EVD) among pregnant women, the rVSV-ZEBOV vaccine clinical trials excluded pregnant women. This impacted access to critical life-saving interventions during the subsequent Tenth EVD epidemic in DRC, when—due to lack of evidence because of the beforementioned exclusions—pregnant and lactating women did not partake in ring vaccination campaigns, until the decision was reversed 10 months later. This not only led to unnecessary mortality of this vulnerable group, but severely impacted women’s right to decide on research participation and community trust in the intervention, just as did the approval of Truvada solely for cisgender males and transgender women [13,14].

The inclusion of sex and gender aspects in drug development and clinical trials is essential, not just for a thorough understanding of efficacy and safety aspects of drugs, but also to ensure there is equity in the distribution of innovation and discovery benefits of COVID-19 therapeutics and vaccines [3]. A group of clinicians, scientists and gender specialists working on global health, sex and gender research and human rights are thus calling for action towards the inclusion of sex and gender, and other socially relevant variables, into the methodology of COVID-19-related trials. Such an approach should become a universal and manifest part of future clinical studies, to allow more personalized patient care and contribute to universal health coverage.

## Appendix A

### Appendix A.1. The Sex and Gender in COVID-19 Clinical Trials Working Group

Evelyne Bischof	Shanghai University
Sabine Oertelt-Prigione	Radboud University Medical Center
Rosemary Morgan	Johns Hopkins Bloomberg School of Public Health
Sabra Klein	John Hopkins Bloomberg School of Public Health
Clare Wenham	London School of Economics and Political Science (LSE)
Nazneen Damji	UN Women
Alison ROWE	UN Women
Laura Mamo	San Francisco State University lmamo@sfsu.edu
Sulzhan Bali	Women in Global Health sulzhan@gmail.com
Susan E. Bell	Drexel University seb376@drexel.edu
Ann Keeling	Women in Global Health
Chandani Kharel	HERD International
Ilana Löwy	CERMES 3, (INSERM, CNRS, EHESS, Paris V)
Anna Coates	Panamerican Health Organization
Shirin Heidari	GENDRO’s Health
Petra Verdonk	Amsterdam UMC-VUmc
Arne Ruckert	University of Ottawa
Luisa Enria	University of Bath
Shelley Lees	London School of Hygiene and Tropical Medicine (LHTM)
Amber Peterman	University of North Carolina
Sean Hillier	York University

### Appendix A.2. Gender and COVID-19 Working Group

Clare Wenham	London School of Economics
Rosemary Morgan	John Hopkins University
Julia Smith	Simon Fraser University
Evelyne Bischof	Shanghai University of Medicine and Health Sciences, Federico II University, Napoli, Italy, and Women’s Brain Project
Sabra Klein	John Hopkins University
Madeline Johnson	Global Affairs Canada
Chris Berzins	Global Affairs Canada
Sulzhan Bali	Women in Global Health
Karen Grepin	University of Hong Kong
Susan Mackay	GAVI
Denise Nacif Pimenta	Oswaldo Cruz Foundation
Niyati Shah	USAID
Kelly Thompson	independent consultant
Sabine Oertelt-Prigione	Radboud University
Amber Peterman	University of North Carolina and UNICEF Innocenti
Ruth Kutalek	Medizinischen Universität Wien
Sophie Harman	QMUL
Ilana Lowy	French National Centre for Scientific Research
Nazeen Damji	UN Women
Ann Keeling	Women in Global Health
Kate Hawkins	Pamoja Communications
Myra Betron	Jhpiego
Susan Bell	Drexel University
Manasee Mishra	IIHMR University, India
Sean Hillier	York University
Yara M. Asi	University of Central Florida
Shelley Lees	London School of Hygiene and Tropical Medicine
Alan White	Leeds Beckett University
Nigel Mxolisi Landa	Great Zimbabwe University
Pavitra Kotini-Shah	University of Illinois at Chicago
Megan O’Donnell	Center for Global Development
Jelke Boesten	King’s College London
Goleen Samari	Columbia University
Alexa Yakubovich	University of Toronto
Liana R Woskie	Harvard Global Health Institute
Peter Baker	Global Action on Men’s Health
Camila Pimentel	Oswaldo Cruz Foundation/Aggeu Magalhães Institute
Derek M. Griffith	Vanderbilt University
Sara Davies	Griffith University
Elena Marbán-Castro	Barcelona Institute for Global Health
Claudia Abreu Lopes	United Nations University, International Institute for Global Health
